# Multisystem Inflammatory Syndrome after SARS-CoV-2 Infection and COVID-19 Vaccination

**DOI:** 10.3201/eid2707.210594

**Published:** 2021-07

**Authors:** Mark B. Salzman, Cheng-Wei Huang, Christopher M. O’Brien, Rhina D. Castillo

**Affiliations:** Kaiser Permanente West Los Angeles Medical Center, Los Angeles, California, USA (M.B. Salzman);; Kaiser Permanente Los Angeles Medical Center, Los Angeles (C.-W. Huang);; Kaiser Permanente Zion Medical Center, San Diego, California, USA (C.M. O’Brien);; Kaiser Permanente Tustin Ranch Medical Offices, Tustin, California, USA (R.D. Castillo)

**Keywords:** multisystem inflammatory syndrome in children, multisystem inflammatory syndrome in adults, SARS-CoV-2, SARS-CoV-2 vaccine, COVID-19, severe acute respiratory syndrome coronavirus 2, viruses, respiratory infections, zoonoses, vaccines, United States, coronavirus disease

## Abstract

We report 3 patients in California, USA, who experienced multisystem inflammatory syndrome (MIS) after immunization and severe acute respiratory syndrome coronavirus 2 infection. During the same period, 3 adults who were not vaccinated had MIS develop at a time when ≈7% of the adult patient population had received >1 vaccine.

Multisystem inflammatory syndrome (MIS) in children (MIS-C) and adults (MIS-A) are febrile syndromes with elevated inflammatory markers that usually manifest 2–6 weeks after a severe acute respiratory syndrome 2 (SARS-CoV-2) infection (*1*–*3*). The Brighton Collaboration Case Definition for MIS-C/A was recently published to be used in the evaluation of patients after SARS-CoV-2 immunization (*3*); some scientists are concerned that vaccination against SARS-CoV-2 can trigger MIS-C/A. We report 6 cases of MIS from a large integrated health system in Southern California, USA; 3 of those patients received SARS-CoV-2 vaccination shortly before seeking care for MIS. All 6 patients met the Brighton Collaboration Level 1 of diagnostic certainty for a definitive case and had MIS illness onset between January 15–February 15, 2021. The Chief Compliance Officer for the Southern California Permanente Medical Group reviewed this case series and confirmed that it was compliant with the Health Insurance Portability and Accountability Act for publication.

## The Study

Patient 1 was a 20-year-old Hispanic woman who sought care for 3 days of a diffuse body rash, tactile fever, sore throat, mild neck discomfort, and fatigue. There was no cough, congestion, headache, or abdominal pain. She had vomiting and diarrhea, which had subsided 8 days before admission. She received her first dose of SARS-CoV-2 vaccine 15 days before admission. She had no known coronavirus disease (COVID-19) exposure but was SARS-CoV-2 PCR and nucleocapsid IgG positive. She was hypotensive at arrival to the emergency department, requiring inotropic support. She had elevated troponin and brain natriuretic peptide (BNP) with a left ventricular ejection fraction initially mildly reduced at 45% but 30%–35% the following day. She responded well to therapy with intravenous immunoglobulin (IVIG) and methylprednisolone (Table 1).

**Table 1:** Demographic, laboratory, and clinical characteristics of 3 patients who had multisystem inflammatory syndrome after SARS-CoV-2 immunization, Southern California, USA

Characteristic	Patient 1	Patient 2	Patient 3
Age, y/sex	20 y/F	40 y/M	18 y/M
Race/ethnicity	Hispanic/Latina	Hispanic/Latino	Asian/Filipino
Underlying conditions	Asthma	Depression, hyperlipidemia	Asthma
Symptoms	Fever and rash for 3 d, diarrhea, vomiting, cardiogenic shock, acute renal failure	6 d of fevers, malaise, diarrhea, neck pain, headache, lethargy	3 d of fever, 2 d of abdominal pain, diarrhea, vomiting and headache
Initial vital signs	Pulse: 130 beats/min, BP 73/56 mm Hg, RR 20 breaths/min, temp 99.4°F, repeat temp 101.4, O2 sats 99% on RA; BMI: 27.85	Pulse 102 beats/min, BP 136/88 mm Hg, RR 20 breaths/min, temp 99.2°F, O2 sats 97% on RA; BMI: 28.89	Pulse 96 beats/min, BP 98/58 mm Hg, RR 20 breaths/min, temp 97.9°F, sats 97% on RA; BMI: 23.99
Treatment	Vasopressors × 3 d, IVIG 100 g, methylprednisolone 1 g/d for 3 d, heparin, broad spectrum antibiotics, remdesivir	Dexamethasone 6 mg/d for 10 d, ceftriaxone, azithromycin, enoxaparin	IVIG 100 g, methylprednisolone 1 g/d for 3 d, anakinra 100 mg/d for 3 d, broad-spectrum antibiotics, aspirin
Imaging	TTE: normal LV, mildly reduced EF 45% which decreased to 30%–35% the next day; chest radiograph: subtle bibasilar ground glass opacities	EKG: ST depression and T wave inversion in inferior leads; TTE: normal LV; EF: 50%–55%; CT angiogram: no pulmonary embolism, minimal ground glass opacities	TTE: normal LV size with mild to moderately reduced EF 40%–45%, right ventricle mildly dilated with normal systolic function; chest radiograph: right pleural effusion; CT abdomen and pelvis: hepatomegaly, splenomegaly, small ascites; pericholecystic fluid; retroperitoneal adenopathy.
Length of hospital stay	8 d	3 d	9 d
First vaccine	12 d before symptom onset	42 d before symptom onset	19 d before symptom onset
Second vaccine	NA	4 d before symptom onset	NA
Previously known COVID-19 disease	No	34 d before symptom onset	43 d before symptom onset
Initial lab results (reference range)			
Serum leukocytes, × 1,000/mcL (4.5–14.5)	32.3	11.3	7
Lymphocytes absolute, × 1,000/mcL (1.5–6.8)	0.55	0.94	0.26
Neutrophils absolute, × 1,000/mcL (1.5–8.00)	31.75	12.68	6.28
Platelets, × 1,000/mcL (130–400)	155	312	63
Creatinine, mg/dL (<1.00)	2.64	1.12	1.12
C-reactive protein, mg/L (<7.4)	378	199.4	185.5
D-dimer, µg FEU/mL (<0.49)	3.01	1.15	3.44
Ferritin, ng/mL (17–168)	533	1,079.7	3,002
Fibrinogen, mg/dL (218–441)	801	875	693
Troponin, ng/mL (<0.03)	1.54	0.37	0.06
BNP, pg/mL (<99)	1,498	672	106
LDH, U/L (<279)	251	156	291
AST, U/L (<34)	43	55	59
ALT, U/L (<63)	28	83	58
Procalcitonin, ng/mL (0.0–0.1)	160.92	0.01	4.41
SARS-COV-2 nucleocapsid IgG qualitative	Positive	Positive	Positive
SARS-COV-2 PCR	Positive	Positive	Negative
Blood culture	Negative × 2	Negative × 2	Negative × 2
Urine culture	Negative	Not done	Negative (after antibiotics)
Bacterial GI PCR panel	Negative	Not done	Negative

*All patients received the Pfizer-BioNTech vaccine (https://www.pfizer.com). ALT, alanine aminotransferase; AST, aspartate aminotransferase; BMI, body mass index; BNP, brain natriuretic peptide; BP, blood pressure; CT, computed tomography; EF, ejection fraction; EKG, electrocardiogram; GI, gastrointestinal; IVIG, intravenous immunoglobulin; LDH, lactate dehydrogenase; LV, left ventricle; MR, mitral regurgitation; NA, not applicable; RA, room air; RR, respiratory rate; SARS-CoV-2, severe acute respiratory syndrome coronavirus 2; sats, saturations; temp, temperature; TR, tricuspid regurgitation; TTE, transthoracic echocardiogram.

Patient 2 was a 40-year-old Hispanic man who sought care after 6 days of episodic fevers up to 101.7°F. Associated symptoms included dyspnea on exertion, headache, neck pain, lethargy, abdominal pain, and diarrhea. No chest pain was present. He had a history of SARS-CoV-2 vaccination and laboratory-confirmed mild to moderate COVID-19, both within 48 days before seeking care (Figure). His exam was notable for sweats, diffuse abdominal pain on palpation, tachycardia, and tachypnea. Patient 2 fulfilled Brighton Level 1 criteria for MIS-A with documented fevers, gastrointestinal and neurologic symptoms, elevated inflammatory and cardiac markers, and electrocardiogram changes that were concerning for myocarditis (*3*). He responded well to treatment with dexamethasone (Table 1).

**Figure:**
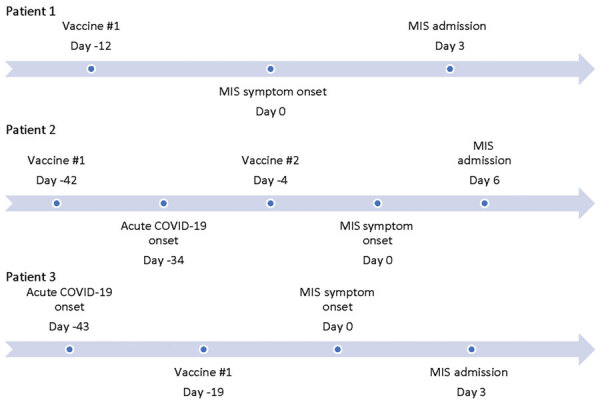
Timeline displaying intervals between coronavirus (COVID-19) vaccine, acute COVID-19 symptom onset, and MIS symptom onset in patients in California, USA. MIS, multisystem inflammatory syndrome.

Patient 3 was an 18-year-old Asian American man who sought care at the emergency department with a history of 3 days of fever as high as 104°F with headache, vomiting, diarrhea, and abdominal cramping (Figure). He denied any upper respiratory symptoms. He had a history of a laboratory-confirmed COVID-19 infection 6 weeks before the onset of symptoms and received the first dose of the SARS-CoV-2 vaccine 18 days before the onset of symptoms. In the emergency department, he was found to be hyponatremic and hypotensive (Table 1). His examination was notable for tachycardia and abdominal tenderness. He had elevated inflammatory markers, thrombocytopenia, and lymphopenia. Echocardiogram revealed mild to moderate reduced systolic function with an ejection fraction of 40%–45%. He responded well to therapy with methylprednisolone, IVIG, and anakinra. 

Patient 4 was a 62-year-old Asian American man who sought care at the emergency department for fever lasting 5 days. For 6 days he had had nausea and vomiting, which developed 23 days after a laboratory-confirmed mild to moderate acute COVID-19 illness that subsided after 1 week. He also had 4 days of bilateral hearing loss. He was hypotensive, requiring inotropic support. He had thrombocytopenia, elevated inflammatory markers, and elevated troponin with diffuse ST elevations on electrocardiogram (Table 2). He responded well to treatment with methylprednisolone, including improvement in his hearing loss.

**Table 2:** Demographic, laboratory, and clinical characteristics of patients who had multisystem inflammatory syndrome without SARS-CoV-2 immunization, California, USA

Characteristic	Patient 4	Patient 5	Patient 6
Age/sex	62 y/M	29 y/F	23 y/M
Race/ethnicity	Asian	Hispanic/Latina	Hispanic/Latino
Underlying conditions	Hyperlipidemia, gout, atrial fibrillation	Obesity	Asthma, obesity
Signs and symptoms	6 d of fever, vomiting, abdominal pain, 4 d of hearing loss; shock, acute renal failure	4 d of fever, headaches, vomiting, abdominal pain; conjunctivitis, shock, acute kidney injury	4 d of fever, abdominal pain, diarrhea, cough, SOB; shock
Initial vital signs	Pulse 121 beats/min, BP 112/63 mm Hg, RR 20 breaths/min, temp 101.6°F, O2 sats 98%; within 1 h in ER: BP 70/56 mm Hg, pulse 112 beats/min, RR 28 breaths/ min, O2 sat 97%; BMI: 28.1	Pulse 140 beats/min, BP 102/71 mm Hg (61/48 mm Hg after 5 h of being in ER), RR 20, temp 105.2°F, O2 sats 99%; BMI: 31.63	Pulse 125 beats/min, BP 87/27 mm Hg, temp 98.2°F, O2 sats 98% on RA; BMI: 40.3
Treatment	Vasopressors, methylprednisolone 125 mg every 6 h, broad spectrum antibiotics, enoxaparin	Vasopressors, methylprednisolone 30 mg every 12 h, IVIG 100 g, heparin, ceftriaxone, ciprofloxacin	Vasopressors, IVIG 2 g/kg, methylprednisolone 1 g daily for 3 d, broad spectrum antibiotics
Imaging	EKG: diffuse ST elevation; TTE: mild concentric LVH, mild LV systolic dysfunction, EF 50%; CT angiogram: no evidence of embolus; increased interstitial markings and hazy ground glass changes, small bilateral pleural effusions; 6 mm pericardiac effusion; ultrasound: right popliteal DVT	TTE: LVEF 50%–55%, mild TR regurgitation, abdominal CT with colitis and enlarged lymph nodes	EKG: sinus tachycardia, no ST changes; TTE: LVEF 20%, global hypokinesis, abdominal CT with mesenteric adenitis
Length of hospital stay	7 d	10 d	12 d; deceased
First vaccine	NA	NA	NA
Second vaccine	NA	NA	NA
Previously known COVID-19	23 days before symptom onset	28 d before symptom onset	38 d before symptom onset
Initial lab results (reference ranges)			
Serum leukocytes, × 1,000/mcL (4.5–14.5)	18.4	10.2	6.8
Lymphocytes absolute, × 1,000/mcL (1.5–6.8)	0.00	0.35	0.52
Neutrophils absolute, × 1,000/mcL (1.5–8.00)	17.66	9.66	14.35
Platelets, × 1,000/mcL (130–400)	102	170	185
Creatinine, mg/dL (<1.00)	2.24	0.78	2.49
C-reactive protein, mg/L (<7.4)	351.7	364.9	246.3
D-dimer, µg FEU/mL (<0.49)	7.21	5.79	>4
Ferritin, ng/mL (17–168)	5,032	606	1,273 at admission, >18,000 at its peak 2 days later
Fibrinogen, mg/dL (218–441)	N/A	N/A	454
Troponin, ng/mL (<0.03)	0.85	0.06	<0.02
BNP, pg/mL (<99)	931	331	228
LDH, U/L (<279)	267	N/A	224
AST, U/L (<34)	38	N/A	42
ALT, U/L (<63)	40	55 8	88
Procalcitonin, ng/mL (0.0–0.1)	Not done	8.15	29.37
SARS-COV-2 nucleocapsid IgG qualitative	Not done	Positive	Not done
SARS-COV-2 PCR	Positive	Negative	Positive
Blood culture	Negative x 2	Negative x 4	Negative x 9
Urine culture	Negative (after antibiotics)	Negative (after antibiotics)	Negative (after antibiotics)
Bacterial GI PCR panel	Not done	Negative	Not done

*ALT, alanine aminotransferase; AST, aspartate aminotransferase; BMI, body mass index; BNP, brain natriuretic peptide; BP, blood pressure; CT, computed tomography; COVID-19, coronavirus disease; DVT, deep venous thrombosis; EF, ejection fraction; EKG, electrocardiogram; GI, gastrointestinal; IVIG, intravenous immunoglobulin; LDH, lactate dehydrogenase; LV, left ventricle; MR, mitral regurgitation; NA, not applicable; RA, room air; RR, respiratory rate; SARS-CoV-2, severe acute respiratory syndrome coronavirus 2; sats, saturations; temp, temperature; TR, tricuspid regurgitation; TTE, transthoracic echocardiogram.

Patient 5 was a 29-year-old Hispanic woman who experienced fever, chills, headache, and nausea 28 days after a laboratory-confirmed acute COVID-19 illness. She sought care at the emergency department with hypotension requiring ionotropic support. Clinicians diagnosed MIS-A on the basis of conjunctivitis, evidence of colitis on abdominal imaging, elevated inflammatory markers, lymphopenia, and elevated BNP. She responded well to treatment with methylprednisolone and IVIG (Table 2).

Patient 6 was a 23-year old Hispanic man who experienced fever and abdominal pain 38 days after a laboratory-confirmed mild to moderate acute COVID-19 illness. He was hypotensive, requiring inotropic support. He had mesenteric adenitis on abdominal imaging. He had elevated inflammatory markers, neutrophilia, lymphopenia, and a left ventricular ejection fracture of 20% on echocardiogram. He was treated with IVIG and methylprednisolone (Table 2). He died 12 days after admission.

## Conclusions

At the time of our study, our medical group was only vaccinating healthcare workers and patients >75 years of age. The 3 patients that were immunized qualified for early vaccination because they either worked or volunteered in a healthcare setting. These cases occurred ≈1 month after the peak surge of COVID-19 cases in Southern California. At the time these patients sought care, only ≈7% of the adult (>18 years of age) population who were members of the Kaiser Permanente patient group (≈3,776,000 members) had received >1 SARS-CoV-2 vaccine, whereas 3 of the 6 patients in this study who had MIS were vaccinated. These 6 patients were hospitalized at 5 of the 15 Kaiser Permanente medical centers across Southern California. We believe the temporal association after SARS-CoV-2 immunization is worth noting, given the theoretical concern of MIS-C/A after vaccination (*3*). We did not identify any patients with MIS after vaccination who did not have recent SARS-CoV-2 infection. It is possible that other case-patients in our member population were hospitalized outside of our 15 medical centers and thus were not captured for this case series.

Overall, MIS is rare in adults. In comparison we treated >50 children with MIS-C during January 2021–February 2021 and >100 since May 2020 among a pediatric population of 960,000.

The Centers for Disease Control and Prevention (CDC) allows for vaccination after a SARS-CoV-2 infection after recovery from the acute illness and after the isolation period, with no recommended minimal interval between infection and vaccination (*4*). Most cases of MIS-C/A occur 2–6 weeks after an exposure or infection (*1*–*3*), although we have seen several children brought for care as late as 8–10 weeks after a confirmed infection or exposure. We need to continue to monitor for MIS-C/A after SARS-CoV-2 infection and immunization as more of the population are vaccinated, especially as vaccines are administered to children who are at higher risk for MIS. CDC and the US Food and Drug Administration co-manage VAERS (the Vaccine Adverse Event Reporting System), which is being used to monitor for adverse events after COVID-19 vaccines. MIS-C/A is listed as a postvaccination adverse event of special interest (*5*) and should be reported to VAERS (*6*).
